# Preoperative Evaluation for Carotid Endarterectomy Using CT and MRI Fusion Images Without Contrast Media

**DOI:** 10.7759/cureus.54321

**Published:** 2024-02-16

**Authors:** Akihito Hashiguchi, Takeshi Tonegawa, Kozo Tashima, Koichi Moroki, Hajime Tokuda

**Affiliations:** 1 Neurological Surgery, Tokuda Neurosurgical Hospital, Kanoya, JPN

**Keywords:** unstable plaque, magnetic resonance angiography, magnetic resonance imaging, carotid plaque, carotid endarterectomy, carotid artery stenosis

## Abstract

The usefulness of carotid endarterectomy (CEA) for carotid artery stenosis has been established even in the era of endovascular treatment. Digital subtraction angiography (DSA) and three-dimensional computed tomography angiography (3D-CTA) are used for preoperative evaluation of CEA; however, contrast agents cannot be used in patients with renal dysfunction or contrast agent allergy. Since the introduction of a three-dimensional image analysis software, SYNAPSE VINCENT (Fujifilm, Tokyo, Japan) in February 2016, we initially fused cervical CT, carotid three-dimensional time-of-flight magnetic resonance angiography, and carotid plaque imaging using 1.5 T magnetic resonance imaging to evaluate carotid artery stenosis in patients with renal dysfunction. Since then, we have gradually accumulated several cases, and at present, this fusion imaging is our first choice for preoperative evaluation of CEA instead of DSA or 3D-CTA. This evaluation method has many advantages over DSA and 3D-CTA, including the fact that it does not require contrast media. We report its usefulness, limitations, and cautions.

## Introduction

The risk of cerebral infarction in carotid artery stenosis has been determined primarily based on morphological assessments such as stenosis degree. However, as determined by ultrasonography, computed tomography (CT), and magnetic resonance imaging (MRI), the quality and quantity of plaque play an increasingly important role in the onset of ischemic events [1]. Although carotid endarterectomy (CEA) is intended to prevent ischemic events, many centers use three-dimensional computed tomography angiography (3D-CTA) and digital subtraction angiography (DSA) because information such as the height and extent of the lesion is needed to evaluate access to the lesion before CEA.

Previously, 3D-CTA was used for the preoperative evaluation of CEA at our institution. In February 2016, we introduced a three-dimensional image analysis system, SYNAPSE VINCENT (Fujifilm, Tokyo, Japan), and evaluated it by fusing these three images of cervical CT, carotid three-dimensional time-of-flight magnetic resonance angiography (3D TOF-MRA), and plaque MRI to replace 3D-CTA. First, we used the fused image for patients with renal dysfunction but found that it had many advantages over 3D-CTA, and now, in principle, CEA is performed based on the fused image.

## Technical report

Symptomatic carotid artery stenosis (>50%) and asymptomatic carotid artery stenosis (>60%) were considered eligible for CEA [2,3]. Preoperatively, patients were evaluated for the presence of systemic atherosclerosis, including coronary artery lesions. All patients underwent microscopic CEA with an internal shunt under general anesthesia while taking antiplatelet medication.

Non-contrast cervical CT (0.5 mm slice; 80-slice CT Aquilion, Canon, Tokyo, Japan), carotid 3D TOF-MRA, and plaque T1-CUBE were uploaded to SYNAPSE VINCENT, and images were constructed. All MR images were acquired using a SignaTM Explore 1.5T MR system (GE Healthcare, Milwaukee, WI, USA). The following were included in the protocol of this study: T1-CUBE (TR, 600 ms; TE, minimum; FOV, 25 x 25 cm; matrix, 256×192; slice thickness, 1.2 mm; acquisition time, 5 min 55 s) and 3D TOF-MRA (TR, minimum; TE, out of phase; FOV, 20 x 25 cm; matrix, 288×160; slice thickness, 2.2 mm; acquisition time, 6 min 19 s).

**Figure 1:**
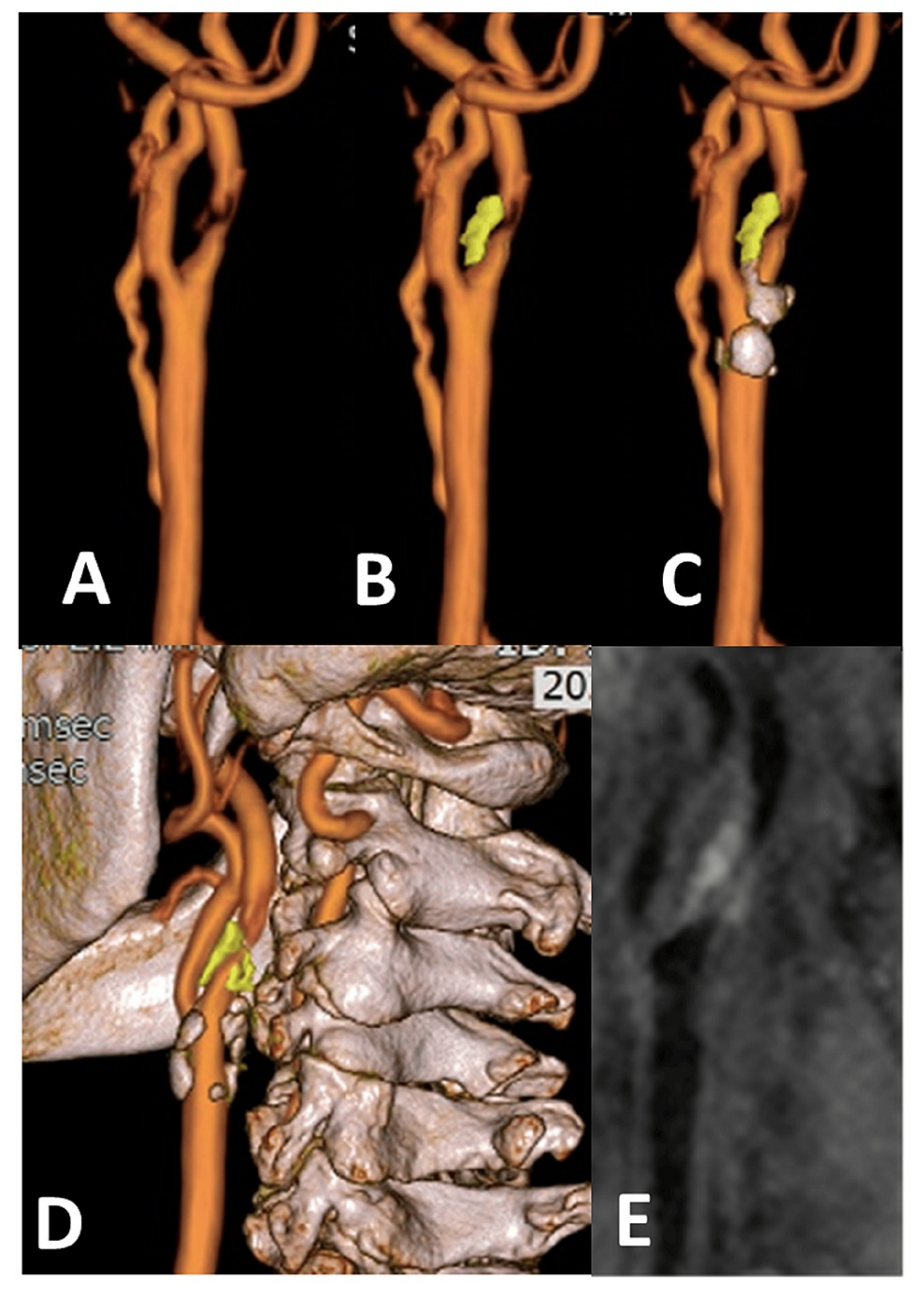
Fused image of a case of symptomatic carotid stenosis on the left A: Carotid three-dimensional time-of-flight magnetic resonance angiography (3D TOF-MRA), showing severe stenosis; B: Superimposed unstable plaque with high signal on T1-CUBE (yellow); C: A calcification component was added (gray); D: Determining whether the distal end of the plaque is accessible or not based on the height of the lesion and the position of the mandible; E: The reconstructed sagittal image of source images of T1-CUBE with fat suppression shows a T1 hyperintense signal

The components of unstable plaques include intraplaque hemorrhage (IPH), lipid-rich necrotic core, thinning or disrupted fibrous capsules, inflammatory cell infiltration, neovascularization, and plaque ulceration [4-6]. Considering the current diagnostic accuracy of MRI, IPH is the most useful for diagnosing unstable plaque in actual clinical practice [4,5,7,8]. In this study, we manually extracted IPH as a component with a higher signal than the sternocleidomastoid muscle (SCM) on T1-CUBE images and used it for fusion images [9]. Fusion of carotid 3D TOF-MRA with unstable components and calcification provides a clear visualization of plaque localization and extent. For evaluation of access to the lesion, images that include bony structures, such as the cervical vertebrae and mandible, are helpful (Figure 1).

Of the 146 CEAs performed at our hospital after introducing SYNAPSE VINCENT until August 2021, 125 (86%) were performed using this fusion image alone, without any problems. Twenty-one patients underwent CEA based on 3D-CTA and eight because the MRA showed a weak perfusion signal at the periphery of the internal carotid artery (ICA) stenosis and was inappropriate for fusion imaging. The remaining 13 patients required 3D-CTA for the simultaneous evaluation of other diseases such as cerebral aneurysms. There was no discrepancy between the preoperative fusion images and intraoperative findings in all 125 (86%) patients who underwent CEA based on the fusion image alone. Intraoperatively, we did not encounter any arteries that were not visualized on the preoperative fusion images. In all cases, the success of CEA was confirmed by imaging studies.

Representative cases

Case 1

**Figure 2:**
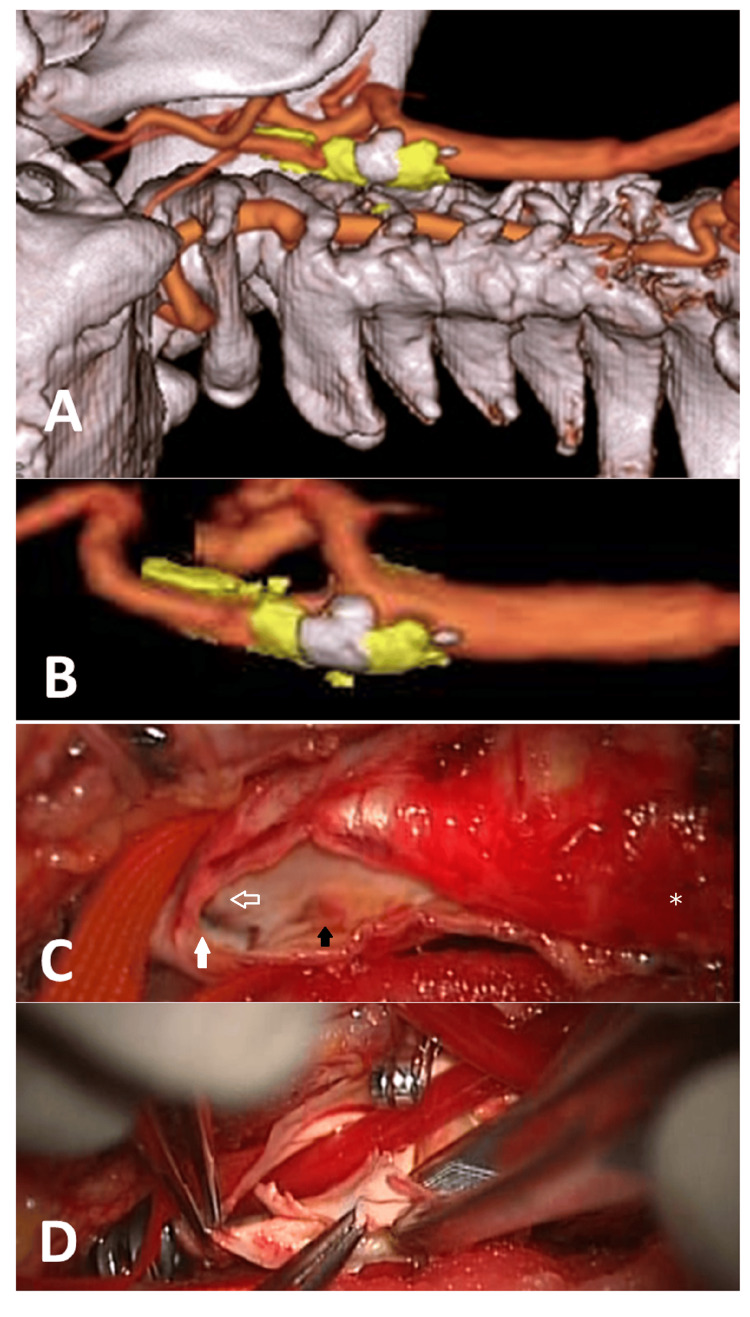
Fusion images and intraoperative findings in Case 1 A and B: The fusion image showed an unstable plaque extending to the kinking of the right internal carotid artery (ICA) at the level of the atlas vertebrae; C and D: Arteriotomy was extended to the kinking in the right ICA (white arrow). A feathered edge of the plaque (white arrow with no fill) was identified. After insertion of the internal shunt, the plaque edge was dissected off from the ICA; Black arrow: intraplaque hemorrhage, *: common carotid artery (CCA)

A man in his 80s underwent CEA for recurrent cerebral infarction despite dual antiplatelet therapy. The distal edge of the carotid plaque was at the level of the atlas vertebra, but the feathered edge of the plaque was also compatible with the preoperative image (Figure 2).

Case 2

**Figure 3:**
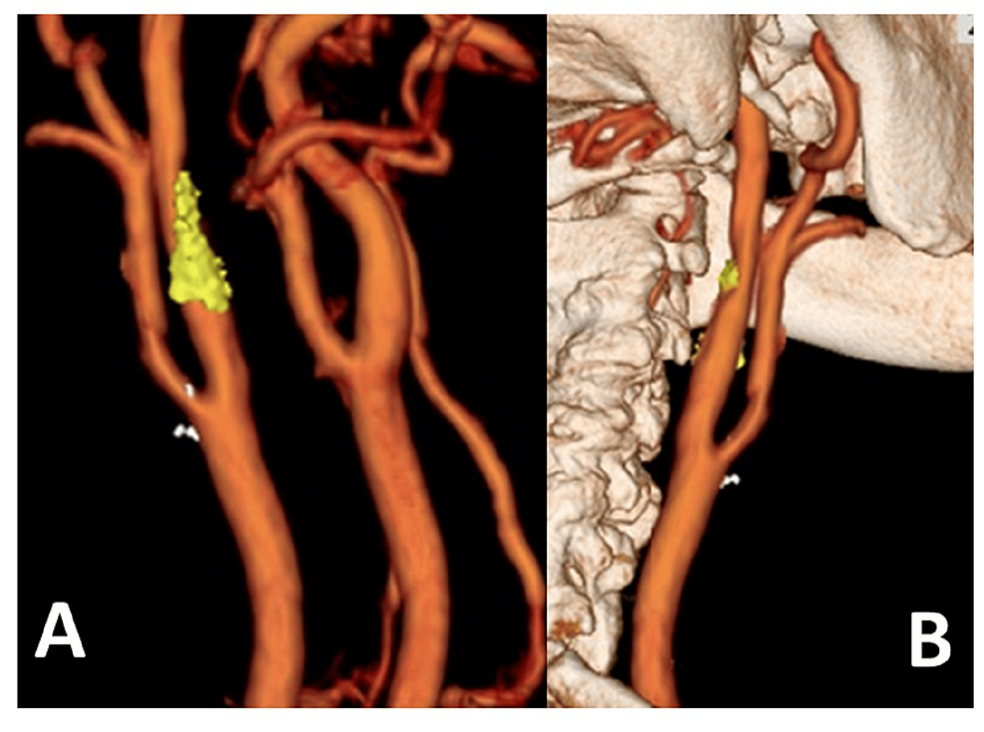
Fusion image of Case 2 A: Unstable plaque is localized distal to the right internal carotid artery (ICA); B: Access to the distal end of the plaque was deemed possible, and carotid endarterectomy (CEA) was performed using an internal shunt as usual.

A man in his 70s underwent CEA for recurrent cerebral infarction while taking dual antiplatelet drugs. The fusion image showed an uneven distribution of unstable plaques on the peripheral side away from the carotid bifurcation (Figure 3), which provided helpful information for clamping the ICA.

## Discussion

The best medical therapy for patients with carotid artery stenosis consists of lifestyle interventions and triple therapy with antiplatelet agents, statins, and angiotensin-converting enzyme inhibitors [10]. In addition, patients with symptomatic carotid stenosis (> 50%) and asymptomatic stenosis (> 60%) are considered for CEA or carotid stenting (CAS) [2,3,10]. However, the appropriate procedure should be selected based on various factors, including anatomy (degree of stenosis, difficulty in accessing CAS or CEA, incomplete Willis ring), past disease and treatment (history of neck surgery or irradiation), and systemic risk (difficulty with general anesthesia) [10]. Although the development of endovascular treatment, including CAS, has been remarkable, the advantage of CEA is that carotid artery stenosis can be improved, and the embolic source can be removed in one stage.

The information required for CEA is the vascular architecture of the common carotid artery (CCA), ICA, branches of the external carotid artery system, and the extent and composition of the plaque itself. 3D-CTA and DSA can provide information on vascular architecture, and these contrast-enhanced examinations are superior in recognizing the exact degree of stenosis. However, although the evaluation of the degree of stenosis is somewhat inaccurate, the ability to depict the carotid artery without using contrast media and the ability to detect high-risk plaques by MR plaque imaging [4,5] and display these superimposed images is valuable preoperative information for the surgeon. To complete CEA, it is crucial to secure the ICA to the peripheral side of the plaque. However, fusion images can quickly identify plaque fragments and calcified components that are unevenly distributed away from the main plaque site and proximal circumferential calcification, which may be a problem during arterial clamping. This information can be important in reducing the risk of complications such as embolism and arterial dissection.

Limitations and cautions

This evaluation method is unsuitable for patients with conditions that make MRI difficult such as those with implanted pacemakers or claustrophobia. This method is simple because SYNAPSE VINCENT semi-automatically fuses the individual CT and MRI test results, but the possibility of misalignment should be recognized. To address this issue, it is essential to confirm that the V3 portion of the vertebral artery is precisely delineated on the vertebral sulcus of the atlas cervical vertebra. Currently, diagnosticians use the T1-CUBE sequence to manually extract plaque components that show a higher signal than SCMs, so the extent to which the plaque is extracted varies among diagnosticians [10]. However, it is expected that artificial intelligence will be able to automatically identify each component and its range within the plaque and fuse the images in the future.

## Conclusions

Using SYNAPSE VINCENT, fusion images consisting of carotid 3D TOF-MRA, MR plaque images, and cervical CT are simple to create and make it easier for surgeons performing CEA to visually recognize essential information. This may be the first examination performed as a preoperative evaluation for CEA, not only for patients who have difficulty using contrast media.

## References

[1] Brinjikji W, Huston J 3rd, Rabinstein AA, Kim GM, Lerman A, Lanzino G (2016). Contemporary carotid imaging: from degree of stenosis to plaque vulnerability. J Neurosurg.

[2] Barnett HJ, Taylor DW, Eliasziw M (1998). Benefit of carotid endarterectomy in patients with symptomatic moderate or severe stenosis. N Engl J Med.

[3] Halliday A, Harrison M, Hayter E (2010). 10-year stroke prevention after successful carotid endarterectomy for asymptomatic stenosis (ACST-1): a multicentre randomised trial. Lancet.

[4] Gupta A, Baradaran H, Schweitzer AD (2013). Carotid plaque MRI and stroke risk: a systematic review and meta-analysis. Stroke.

[5] Gupta A, Baradaran H, Kamel H (2014). Intraplaque high-intensity signal on 3D time-of-flight MR angiography is strongly associated with symptomatic carotid artery stenosis. AJNR Am J Neuroradiol.

[6] Smith LC, Funnell JP, Richards T, Best LM (2023). Carotid plaque ulceration: unquantified predictor of stroke. BJS Open.

[7] Gupta A, Mushlin AI, Kamel H, Navi BB, Pandya A (2015). Cost-effectiveness of carotid plaque MR imaging as a stroke risk stratification tool in asymptomatic carotid artery stenosis. Radiology.

[8] Wasserman BA, Astor BC, Sharrett AR, Swingen C, Catellier D (2010). MRI measurements of carotid plaque in the atherosclerosis risk in communities (ARIC) study: methods, reliability and descriptive statistics. J Magn Reson Imaging.

[9] Yoshida K, Narumi O, Chin M (2008). Characterization of carotid atherosclerosis and detection of soft plaque with use of black-blood MR imaging. AJNR Am J Neuroradiol.

[10] Saito A, Sasaki M, Ogasawara K (2012). Carotid plaque signal differences among four kinds of T1-weighted magnetic resonance imaging techniques: a histopathological correlation study. Neuroradiology.

